# Strength and Microscopic Mechanism of Cement–Fly Ash–Slag–Desulfurization Gypsum Solidified Mica Schist Weathered Soil

**DOI:** 10.3390/ma16216957

**Published:** 2023-10-30

**Authors:** Yunzhi Shang, Zhenglong Cui, Yongjing Li, Yannian Zhang, Yaohui Cheng

**Affiliations:** 1 School of Civil Engineering, Liaoning Technical University, Fuxin 123000, China; syzddjs@126.com (Y.S.); lyjsdyt@126.com (Y.L.); cyhddfc@126.com (Y.C.); 2 School of Civil Engineering, Shenyang Jianzhu University, Shenyang 110168, China; zyntiger@163.com; 3 School of Civil Engineering and Transportation, Permafrost Institute, Ministry of Education Northeast-China Station of Permafrost Geo-Environment, Northeast Forestry University, Harbin 150040, China

**Keywords:** mica schist weathered soil, solid waste, strength, microscopic characteristics

## Abstract

Mica schist weathered soil possesses a number of poor engineering characteristics, which make it difficult to use as a subgrade material for resource utilization. Therefore, in this study, a new type of curing agent, CFSD (cement–fly ash–slag–desulfurized gypsum), is proposed for this soil. The effects of different curing agent dosages, age of preservation, and confining pressure on the stress–strain curves were analyzed via the uniaxial compression test and triaxial compression test, while the micromorphological characteristics of cured soil were analyzed via X-ray diffraction analysis and the SEM test combined with Image J software. In this paper, we also establish a microscopic mechanism model to determine how curing agents increase the strength of mica schists. The results reveal that the compressive strength of solidified soil increases rapidly within 28 days; the CFSD dosage of 4% at 7 d increased by 103.23% by 28 d. After 28 d, the trend of compressive strength growth was flat. The CFSD dosage of 4% at 7 d increased by 128.34% by 90 d; with the increase in the dosage, the curve transformed from flat to steep. These results suggest that the CFSD dosage is positively correlated with the damage strain and damage bias stress of solidified soil. The curves for the strain softening type with a 4% dosage as the initial effective confining pressure increased from 50 kPa to 300 kPa; the failure stress and failure strain increased by 202.09% and 90.85%, respectively. With the increase in curing agent dosage and maintenance age, the pore size of 2~5 μm, >5 μm interval decreased from 56.46% to 27.92%, the porosity decreased from 12.51% to 4.6%, and the hydrate produced by the curing agent cemented and filled up the pore space between the loose particles of the soil body. Thus, the large pore space became microporous, and the pore structure densification was greatly improved.

## 1. Introduction

Inorganic binders such as cement and lime are commonly used as solidifying materials in soil. Approximately 510.7 kg of CO_2_ is produced for every 1 t of cement [1]. During the calcination of limestone into lime, over 40% of CO_2_ is liberated in the atmosphere, and the gases and particles emitted during the production process can pollute the environment and affect people’s health [2,3,4,5]. Therefore, it is crucial to find a cementing material with good performance and low cost. At the same time, based on the needs of production and daily life, people’s demand for industrial products is increasing yearly, and the production of industrial solid waste piles continues to rise. The average annual storage and disposal amount is more than 500 million tons, and the total accumulation amount has exceeded 10 billion tons [6]. As such, achieving efficient utilization of industrial solid waste is an urgent problem that has yet to be resolved [7,8].

If a new type of soil solidification agent can be developed based on solid waste materials, this will not only solve the current technical difficulties associated with cement or lime solidification of soil but also the problem of solid waste treatment, which has important practical significance [9,10]. Many scholars have conducted extensive research on this topic. Wu et al. [11] used cement composite admixtures to study the solidification of silt, studied the influence of different admixtures on the stability of soil mechanics characteristics and microstructure, and finally determined that the optimal mixing ratio of fly ash, clay, and gypsum is 4%, 4%, and 2%. Adhikari et al. [12] used fly-ash-based geopolymers to improve medium- and high-plastic soils. Based on the experimental results, regression models were developed, and sensitivity analysis was conducted. The study showed that solidified soil exhibited good performance under both static and dynamic loads. Ren et al. [13] addressed the high energy consumption of traditional thioimidate cement production materials by replacing original materials with high-temperature calcination industrial waste residue; subsequently, the UCS of improved soil reached 7.5 MPa in 28 days. Wang et al. [14] developed a green and environmentally friendly cementitious material based on metakaolin for the solidification of clay, and the results showed that the optimal mixing ratio of alkali activator and metakaolin was 1:2. Sukprasert et al. [15] studied the mechanical and microstructural properties of silty clay modified with a fly-ash-based polymer and analyzed the effects of the FA: BFS replacement ratio, sulfurization temperature, and alkali solution concentration on the UCS of solidified soil. The optimal ratio of FA:BFS was 2:1. Dhar et al. [16] developed a soil amendment based on plastic fibers and lime and conducted solidification improvement tests on clay. The results showed that fibers help increase the elastic modulus, transforming brittle failure into ductile failure. Ebailila et al. [17] studied the effect of the gypsum level on the long-term performance of lime-stabilized sulfate-containing soil and found that the optimal improvement effect was achieved when the ratio of gypsum to lime was 1.5. Zhang Dingwen et al. [18] developed a soil amendment based on industrial solid waste materials, studied the solidified and improved clay, and revealed the changes in the UCS and pH of the improved soil with different dosages and curing times. Yaghoubi et al. [19] studied the improvement of soft marine clay with the addition of an FA- and slag-based polymer and analyzed the influence of preparation factors such as water content, temperature, and curing time. Tests showed that the strength of the improved soil remained essentially unchanged after six dry–wet cycles. Mineralogy analysis found that more C-N-A-S-H (calcium, sodium, aluminum, silicate, hydrate) products were generated. Fang Xiangwei et al. [20] developed a GT (high-calcium ash–gypsum)-type soil conditioner based on high-calcium ash and gypsum. Mechanical and durability tests were conducted on improved soil samples. The results showed that its various properties were significantly improved compared to unmodified soil. Liu et al. [21] prepared a composite cementitious agent composed of cement, steel slag, and metakaolin to stabilize and reinforce soft clay. The results showed that the improved soil exhibited similar properties to cementitious soil. They proposed a new indicator of free water content, which can effectively characterize the compressive strength of solidified soil. Yaghoubi et al. [22] attempted to prepare a new type of improver by mixing industrial by-product fly ash and slag and found that increasing the amount of slag had a significant impact on the development of improved soil strength. The greatest improvement was achieved when the ratio of Na_2_SiO_3_ to NaOH was 7:3. Zhou Cuiying et al. [23] conducted experimental research on red weathered soil and its improvements using self-made disintegration testing equipment. The results showed that the disintegration rate of the sample was closely related to the mineral composition and hydration characteristics, and ester materials can effectively strengthen the cementation effect of the improved soil. Phumiphan et al. [24] utilized fly-ash-based polymers to improve and dispose of red soil and analyzed the effects of various factors on the strength development of the improved soil through a combination of macro- and microresearch methods. Many scholars have carried out systematic research on poor-quality fillers such as soft soil, silt, soft soil foundation, and silty soil, but there are relatively few studies that have explored the solidification of weathered rock materials. If solid waste materials are used to solidify and improve mica schist weathered soil, this will not only save costs but will also be beneficial to environmental protection and will definitely generate significant social and economic benefits.

Therefore, this article proposes using cement–fly ash–slag–desulfurization gypsum to produce a new type of solidification agent, CFSD. It describes the uniaxial compression tests and triaxial compression tests performed on mica schist strongly weathered soil improved with the curing agent CFSD; studies the changes in the stress–strain curve of the solidified soil under different curing agent dosages, ages, and confining pressures; and analyzes the formation mechanism of the strength of the solidified soil through X-ray diffraction analysis and SEM scanning mirror images, providing a theoretical basis for the engineering application of the new soil curing material, CFSD.

## 2. Test Materials and Methods

### 2.1. Test Material

**Figure 1:**
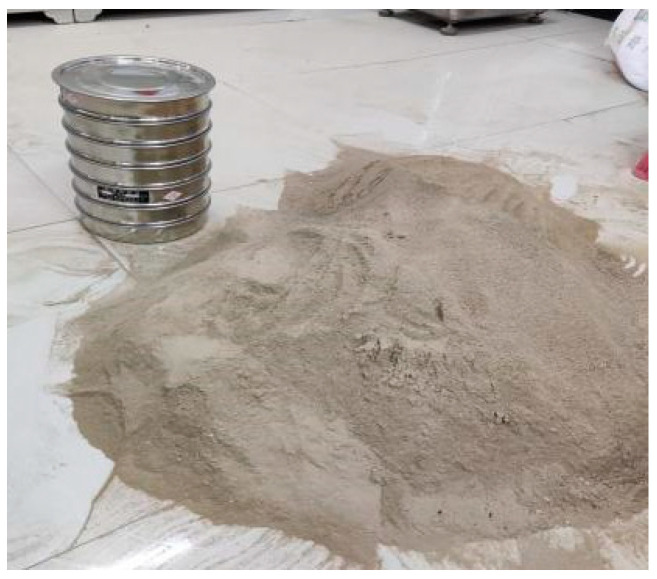
Mica schist weathered soil sample.

**Figure 2:**
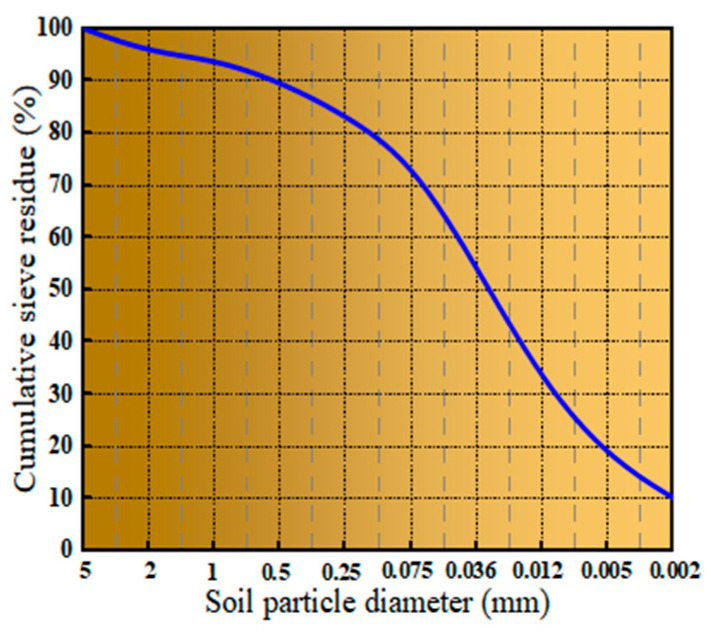
Cumulative particle size distribution.

Figure 1 depicts a sample of soil formed via weathering of mica schist. This sample was taken from the site of a highway construction project in Hubei Province. It has a high degree of weathering and a yellow surface. The particle size distribution is shown in Figure 2. The basic physical properties are shown in Table 1.

**Figure 3:**
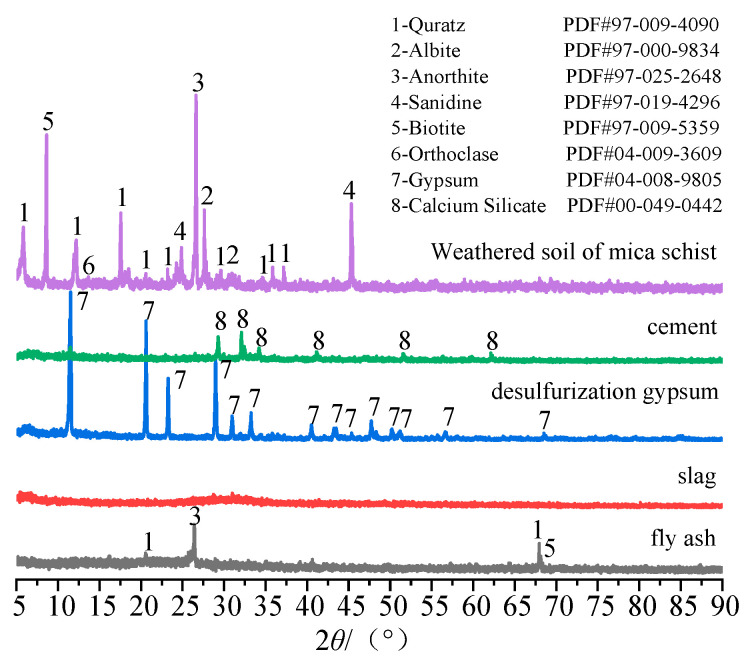
X-ray diffraction patterns of raw materials.

After the preliminary optimization of the ratio, the proportion of each component of the curing agent was determined to be cement:fly ash:slag:desulfurization gypsum = 45:22:17:16. The reaction activity of each component was the highest, and the curing effect was the best. In the composition of the CFSD curing agent, the cement is P.O 42.5 ordinary Portland cement, the fly ash is Class I fly ash, the slag is S95 grade slag, and the gypsum is desulfurization gypsum. The main chemical components of each component are shown in Table 2. The X-ray diffraction patterns of the raw materials are shown in Figure 3.

### 2.2. Test Method

#### 2.2.1. Sample Preparation

**Figure 4:**
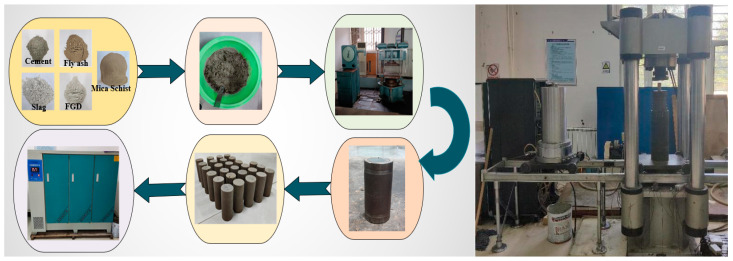
Sample preparation and test equipment.

We screened the soil sample. Next, we mixed the hardener with the soil sample according to the ratio, added the appropriate amount of water to the soil mixture, and mixed it well. The mixture was added to the steel membrane coated with lubricating oil on the inner wall. The test block was compacted and shaped using a pressure testing machine, and the specimen was demolded and sampled after 2 h of resting. After that, the cured soil specimen was placed in a standard curing room to be maintained for the specified duration. The sample preparation and test equipment are shown in Figure 4.

#### 2.2.2. Mechanical Properties

According to JTG 3430-2020 [25], the unconfined compressive strength and shear strength of solidified soil were tested. The unconfined compressive test adopts dimensions of Φ50 mm × 100 mm. The cylindrical specimen was subjected to uniaxial compressive testing on solidified soil specimens with different amounts of curing agents after 28 days of standard curing. The triaxial compression test was carried out using cylindrical specimens with dimensions of Φ50 mm × 100 mm, and after standard maintenance for the specified duration, the triaxial consolidation undrainage test was carried out using a variety of confining pressures, such as 50 kPa, 100 kPa, 200 kPa, and 300 kPa, for the specimens with different amounts of curing agent, respectively.

#### 2.2.3. Microanalysis

The damaged mechanical performance test sample was placed in a 40 °C oven and dried into a powder as if it were a dry sample. According to the test requirements, the sample was treated to a length, width, and height of about 1 cm and then sprayed with gold for SEM analysis and testing. Next, the dried sample was ground in a mortar and passed through a 200-mesh sieve, and a sufficient amount of sample was selected for XRD diffraction analysis. The instrument test range is 5–90°, the scanning speed is 5°/min, the step size is 0.02°, the anticathode is Cu, and the wavelength is 0.154056 nm. The SEM images of the samples were imported into Image J (Image J 1.X) software, and the SEM images were binarized to extract the geometric parameters of the soil pore microstructure. Finally, the parameters of statistical porosity, morphology ratio, and equivalent diameter were calculated for microstructural analysis.

## 3. Test Results and Analysis

### 3.1. Uniaxial Compressive Test

#### 3.1.1. The Effect of Curing Agent Dosage on Stress–Strain of Solidified Soil

**Figure 5:**
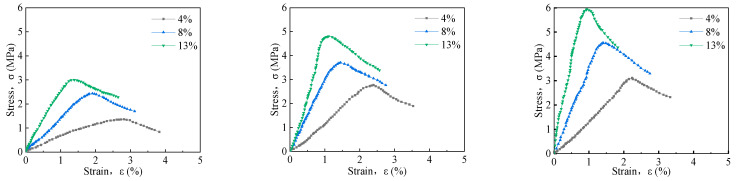
Uniaxial stress–strain curve of 7 d, 28 d, and 90 d solidified soil from left to right.

Figure 5 shows the uniaxial stress–strain curve of solidified soil specimens with curing ages of 7 d, 28 d, and 90 d. As illustrated by the figure, the stress of the solidified soil specimen initially increases before decreasing with the increase in strain. With the increase in the amount of curing agent, the peak stress of the solidified soil specimen shows a positive growth trend, the initial elastic modulus increases, and the stress–strain curve changes from a gentle to a peak shape. When the specimen reaches peak stress, the curve drops sharply, showing a strain-softening behavior. As the amount of curing agent increases, the failure strain corresponding to the peak stress gradually decreases. Taking the curing age of 28 days as an example, with a 4% dosage as the reference value, the failure strain of 8% and 13% dosage decreases by 39.32% and 53.74%, respectively. The brittleness of the solidified soil is significantly enhanced. The failure stress of solidified soil increases by 33.99% and 73.59%, respectively, indicating that with the increase in the amount of solidified agent, the soil structure becomes denser, significantly increasing the peak stress of weathered mica schist soil. This is because with the increase in the dosage of the curing agent, the content of reactive SiO_2_ and Al_2_O_3_ in the soil increases, and the volcanic ash reaction occurs in the alkaline environment provided by the cement, generating a series of hydrates, such as C-S-H (calcium–silicate–hydrate), C-A-H (calcium–aluminate–hydrate), AFt (ettringite), etc., which gel-fill the pore spaces of the soil. As a result, the densification of the soil is enhanced, which significantly increases the peak stresses of the weathered soil with mica schist.

#### 3.1.2. The Effect of Curing Age on Stress–Strain of Solidified Soil

**Figure 6:**
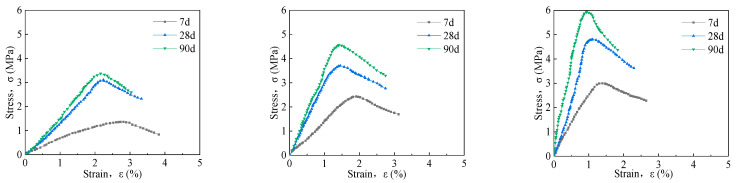
Uniaxial stress–strain curve of 4%, 8%, and 13% solidified soil from left to right.

Figure 6 shows the stress–strain curve of solidified soil with 4%, 8%, and 13% of the content of the curing agent. As shown in the figure, the stress–strain curve of 7 d and 28 d differs significantly, while the stress–strain curve changes slightly at 28 d and 90 d. This indicates that the curing agent greatly improves the early strength of weathered soil of mica schist, while the later strength increases at a slower rate. Taking the solidified soil with 4% of the curing agent as an example, the failure stresses of 7 d, 28 d, and 90 d are 1.362 MPa, 2.768 MPa, and 3.11 MPa, and the failure strains are 2.81%, 2.39%, and 2.24%, respectively. At 7 days, with the increase in curing age, the failure stress increases by 103.23% and 128.34%, respectively, and the failure strain decreases by 14.94% and 20.28%, respectively. This indicates that as the curing age increases, the chemical reactions of each component of the curing agent become more thorough, and the brittleness of solidified soil shows an increasing trend. The strength of the specimen is enhanced over time and continues to increase, rising quickly in the early stages, while the later increases are more gradual. This is because as the sample ages, the curing agent components of the chemical reaction are enhanced, and the strength of the increase is more significant. As the curing components react with the cured soil system, the water content is constantly reduced, and the rate of chemical reaction that promotes soil hardening slows down and levels off over a longer maintenance period. The test data are shown in Table 3.

### 3.2. Triaxial Compression Test

#### 3.2.1. Effect of Confining Pressure on Triaxial Stress–Strain Characteristics

**Figure 7:**
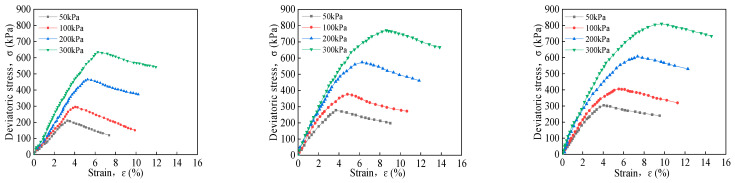
Triaxial stress–strain curve of solidified soil under different confining pressures with 4, 8, and 13% contents from left to right.

Figure 7 shows the stress–strain curve of solidified soil with different amounts of curing agent under different confining pressures. The stress–strain curves of the cured soil are all of the strain-softening type. As the confining pressure increases, the failure deviator stress and failure strain also increase, and the residual strength shows an upward trend. The stress–strain curve peak state is obvious under the confining pressure of 50 kPa and 100 kPa. As the strain development reaches its peak, the stress–strain curve steepens. When the confining pressure is 200 kPa and 300 kPa, the peak of the stress–strain curve is gentle, showing a softening trend. Taking the solidified soil with a curing agent blending ratio of 4% as an example, when the initial confining pressure of the specimens increases from 50 kPa to 100 kPa, 200 kPa, and 300 kPa, the failure deviatoric stress of the solidified soil specimens increases by 40.4%, 121.25%, and 202.09%, respectively. The failure strain of solidified soil specimens increases by 23.17%, 60.06%, and 90.85%, respectively. This is due to the increase in confining pressure, which correspondingly increases the amount of lateral restraint force on the sample, making the soil more compact.

#### 3.2.2. The Effect of Curing Agent Dosage on Triaxial Stress–Strain Characteristics

**Figure 8:**
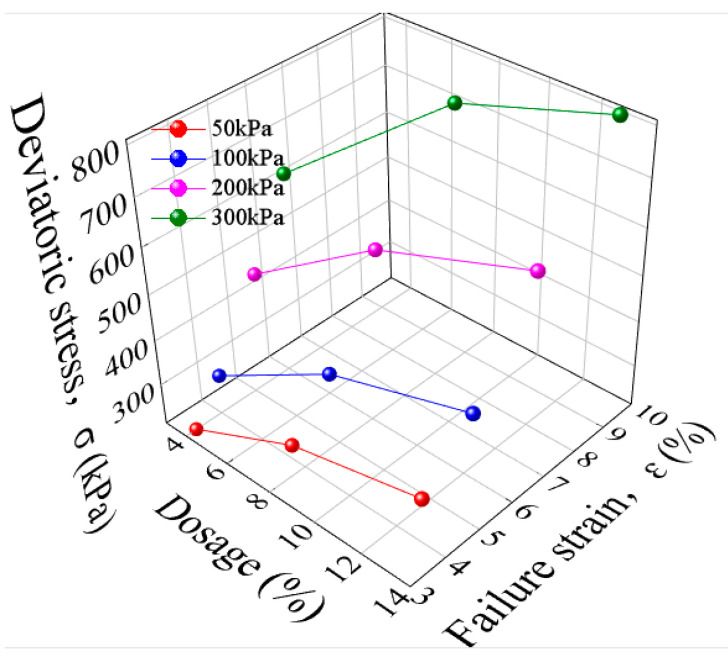
Relationship curve between curing agent and failure deviator stress and failure strain.

Figure 8 shows the stress–strain relationship curves of mica schist solidified soil specimens under different curing agent dosages. The failure strain increases with the increase in the curing agent dosage, and there are notable differences in the growth rate of failure strain under different curing agent dosages. When the confining pressure is 300 kPa and the curing agent dosage is 4%, the failure strain of the solidified soil specimen is 6.26%. When the curing agent dosage is increased to 8% and 13%, the failure strain increases by 37.85% and 55.11%, respectively. Taking the confining pressure of 50 kPa as an example, as the curing agent dosage increases from 4% to 13%, the failure bias stress develops from 210.269 kPa to 304.38 kPa. Based on the dosage of 4%, the failure bias stress of 8% and 13% increases by 32.36% and 44.74%, respectively. This demonstrates that the failure deviator stress of the solidified soil is significantly increased with the amount of curing agent. This is because the chemical components of the curing agent promote each other to generate a gel product with a cementing effect. As a result, the pore space is constantly filled, so the soil structure becomes denser, improving the macro-performance of the peak strength of the soil body. The test data are shown in Table 4.

### 3.3. Microscopic Testing Analysis

#### 3.3.1. Analysis of X-ray Diffraction Results

**Figure 9:**
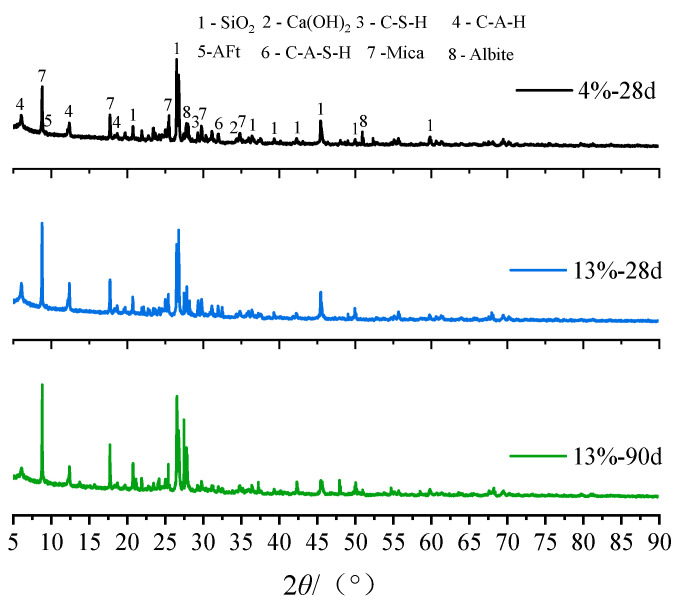
X-ray diffraction pattern.

To investigate the composition of hydration products in mica schist solidified soil, X-ray diffraction tests were conducted on solidified soil samples with different curing agent dosages and curing ages. The XRD pattern is shown in Figure 9. As shown in the figure, the phase composition of the mica schist solidified soil sample is mainly composed of mica (PDF#01-074-6686), ettringite (PDF#01-073-6239), hydrated calcium silicate (PDF#00-033-0306), and hydrated calcium aluminates (PDF#04-016-6492). The diffraction peak intensity of various chemical products in the 4%-28 d solidified soil specimens is relatively weak, and the amount of cementitious material remains low. The content of the hydrated phase in the 13%-28 d solidified soil specimens increases significantly, demonstrating that the reaction rate of active components is significantly strengthened with the increase in the amount of curing agent. The 13%-90 d solidified soil samples show that the intensity of the ettringite diffraction peak and the hydrated calcium aluminate diffraction peak increases significantly with the prolongation of the curing time. This is because the active substances in fly ash and slag are fully activated in the alkaline environment, and the amount of hydration products is further increased. At the same time, the action of the SO_4_^2−^ ion promotes the production of ettringite, which is consistent with the change law of the macromechanical properties of solidified soil.

#### 3.3.2. Analysis of the Mechanism of Strength Formation of Solidified Soil

**Figure 10:**
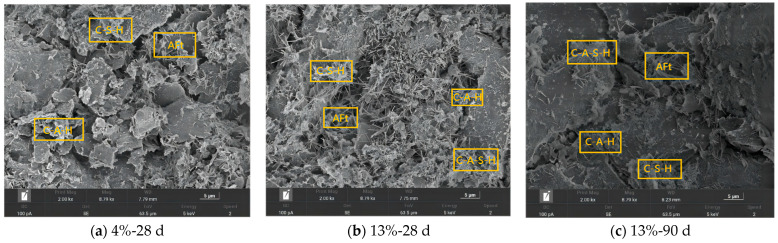
SEM images of solidified soil under different curing agent dosages and curing ages.

The 2000× scanning results of solidified soil samples under different curing agent dosages and curing ages are shown in Figure 10. The scanning electron microscope image shows that after the addition of the CFSD curing agent, a series of chemical reactions occur in the soil, generating various hydrates. As the hydration products develop and grow between the soil pores, the compactness of the soil structure is enhanced, and the macroscopic performance significantly enhances the mechanical properties of the solidified soil specimen [26,27].

The early hydration reaction of 4%-28 d samples in Figure 10a shows that a certain degree of hydration reaction has occurred in the solidified soil, and a small amount of flocculent C-S-H gel, flaky C-A-H gel, and needle stick ettringite are generated, but there are many gaps between soil particles, which are still in a dispersed state. Figure 10b shows that the increase in the amount of curing agent further improves the variety and number of hydration products. The expansive hydration products ettringite and calcium sulfate form a framework by overlapping and interlacing each other in the soil, supporting adjacent soil particles and filling pores. The C-S-H and C-A-H gel in the solidified soil grow in clusters whilst also producing dense reticular structures of hydrated calcium aluminates (C-A-S-H), whose particles are intertwined with each other. The soil particles gradually condense, and the surface structure of the soil becomes more compact. As shown in Figure 10c, the distribution area of hydration products in the soil continuously diffuses with the increase in curing age. The hydration products firmly wrap the soil particles while further filling their pores, and the compactness of the soil is improved.

#### 3.3.3. Microquantitative Analysis

**Figure 11:**
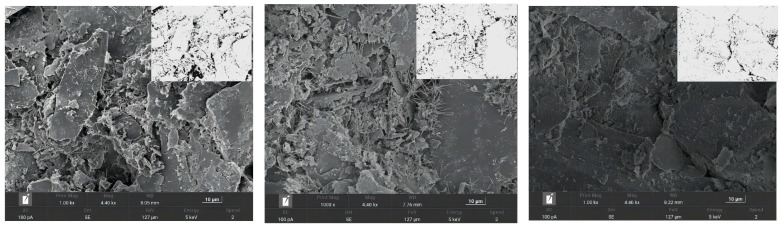
Scanning electron microscopy and binary electron microscopy of solidified mica schist soil.

In order to quantitatively describe the microstructure evolution of solidified soil, Image J software was used to binarize the microscopic morphology electron microscopy images of solidified soil at different ages with different amounts of curing agents under a 1000× microscope. The microscopic pore parameters of solidified soil specimens were extracted for quantitative analysis [28,29,30]. Figure 11 shows the scanning electron microscopy image of mica schist; the SEM image after binarization is presented in the upper right corner.

**Figure 12:**
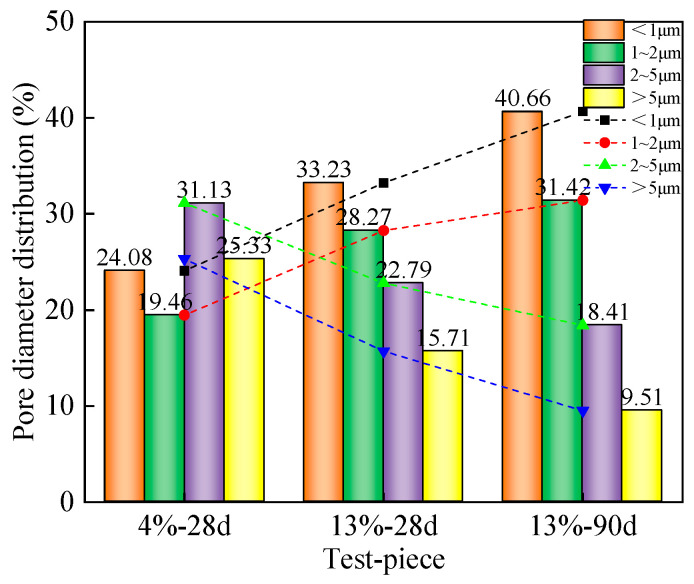
Statistical diagram of micropore diameter distribution of mica schist solidified soil specimens.

Figure 12 shows the statistical map of pore size distribution, which is calculated using an equivalent diameter; the number of holes considered is over 1000. The map is divided into <1 μm according to the pore size of the test piece, as follows: 1~2 μm, 2~5 μm, and >5 μm, the last of which has four intervals. Figure 11 shows that the pore size distribution in the 4%-28 d solidified soil specimen is uneven. At 2~5 μm and >5 μm, the proportion of the interval is the highest, at 56.46%. As the curing agent dosage increases from 4% to 13%, the large pore size distribution decreases while the small pore size distribution increases, within the range of 2~5 μm. At >5 μm, the proportion decreased by 31.81%, <1 μm. At 1~2 μm, the proportion of the interval increases by 40.78%. This indicates that with the increase in curing agent dosage, the generation of hydrates also increases. Hydrates fill the pores of soil, compress the pore space, and improve the large pore size [31]. As the curing age increases from 28 days to 90 days, it can be observed that the pore distribution structure changes with the increase in curing age (μm). At 1~2 μm, the proportion of the interval increases by 17.2%, 2~5 μm. At >5 μm, there is a reduction of 27.48% in the interval. As the curing age increases, the pore size distribution also improves, the amount of contact between particles significantly increases, and the soil structure becomes more compact. Large pores are filled with hydrates and become small pores, optimizing the soil pore structure.

**Figure 13:**
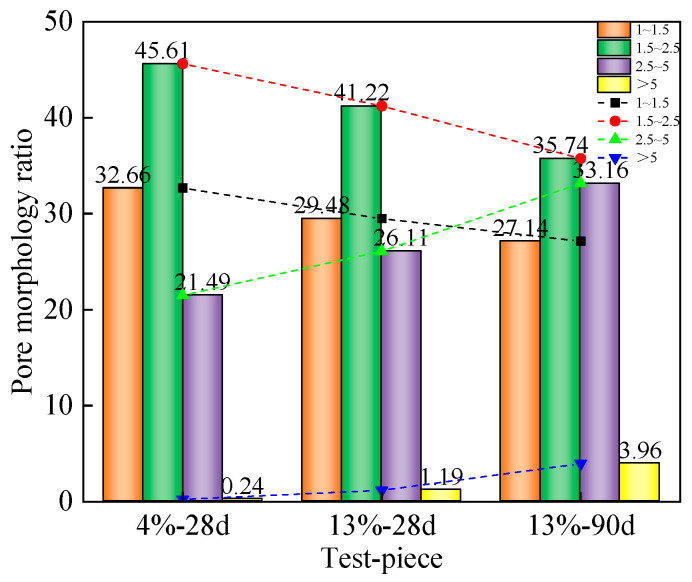
Distribution of microscopic pore morphology ratio of mica schist solidified soil specimens.

Figure 13 shows the distribution map of the micropore morphology ratio of mica schist solidified soil specimens. The pore morphology ratio is the ratio of the long axis to the short axis of the pores, which is closer to one and closer to a circular shape. Conversely, it tends to be more elongated in shape. As shown in the figure, the pore morphology ratio of solidified soil specimens is the most distributed in the range of 1.5–2.5, followed by the range of 1–1.5 and 2.5–5, and is the least distributed in the range of >5. Therefore, the pore morphology is mainly nearly elliptical, elongated, and nearly circular. The proportion of pore morphology ratios of 4%-28 d solidified soil specimens with a ratio of 1–1.5 and 1.5–2.5 is 32.66% and 45.61%, respectively. When the dosage of the curing agent increases from 4% to 13%, the distribution frequency of pore morphology ratios of solidified soil specimens decreases to 29.48% and 41.22%, respectively. With the increase in the curing agent, the pore morphology of solidified soil specimens changes from nearly circular to nearly elliptical and elongated pores. This is mainly because, with the increase in curing agent dosage, more hydration products are generated, which increases the bonding effect between particles, further strengthens the connection between particles, and changes the pore microstructure. As the curing age increases from 28 days to 90 days, the ratio of the pore morphology of solidified soil specimens increases from 26.11% and 1.19% to 33.16% and 3.96%, respectively. The increase in the curing age enhances the chemical reaction of the curing agent, the production of needle bar ettringite and calcium sulfate hydrate generated by hydration increases, and the crystal extends to the pores, playing the role of microfiber between the soil, as well as hydrated calcium aluminates. The cementation and filling effect of gel products such as hydrated calcium silicate significantly improve the distribution of macropores between soil particles and refine the pore structure. The pore morphology gradually evolves from a near-circular shape to a strip shape, and the soil microstructure becomes more compact [32].

**Figure 14:**
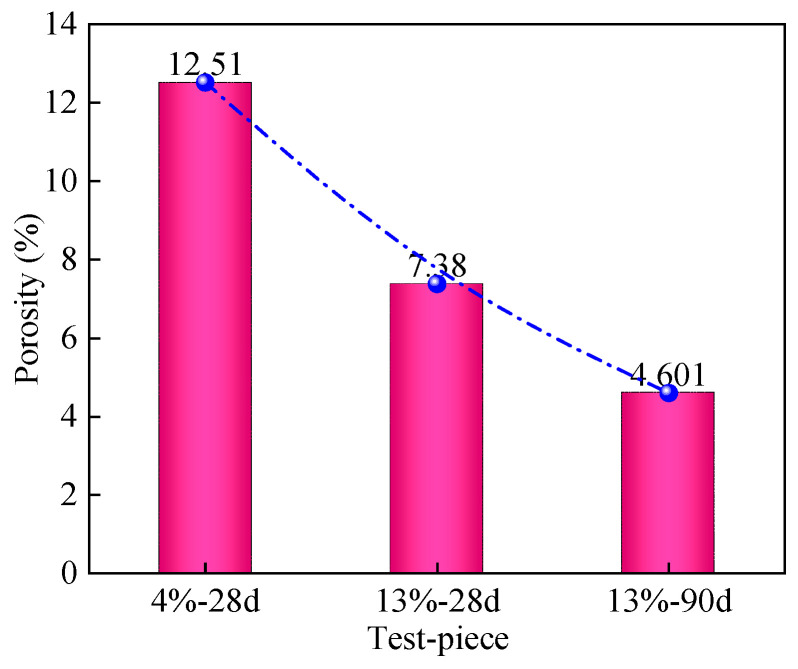
Two-dimensional plane porosity of mica schist solidified soil.

Figure 14 shows the two-dimensional plane porosity of mica schist solidified soil specimens under different curing agent dosages and curing ages. As shown in the figure, the porosity of the solidified soil specimens after 4%-28 days remains relatively high. When the curing agent dosages increase from 4% to 13%, the porosity decreases from 12.51% to 7.38%, which is 41.01% lower than that of the solidified soil specimens after 4%-28 days. The porosity is significantly decreased and further decreases with the increase in curing age. The porosity of the 13%-90 d solidified soil specimen is 4.6%, which is 37.67% lower than that of the 13%-28 d solidified soil specimen. This is mainly because the content of cementitious compounds generated by the curing agent increases with the increase in the dosage of the curing agent, causing soil particles to quickly aggregate and the system to connect more tightly. With the increase in curing age, the amount of needle-like crystals generated increases, forming a dense interlocking structure, and the density of the microstructure is increased, resulting in a gradual decrease in porosity [33].

## 4. Discussion of the Microscopic Mechanism of Improved Mica Schist Weathered Soil

**Figure 15:**
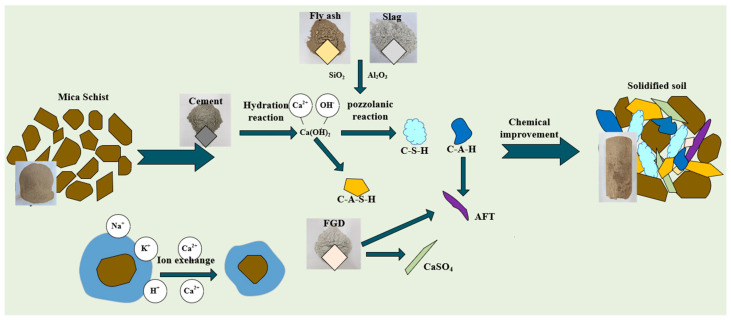
Schematic diagram of the microscopic mechanism model of CFSD improved strongly weathered mica schist.

Figure 15 shows the microscopic mechanism model of CFSD combined improvements of strongly weathered mica schist. The figure shows that the strength formation of mica schist improved soil samples comes from the cementitious filling effect of new curing agent chemical products. The weathered mica schist soil filler is significantly affected by the binding water, and the proportion of highly valent cations in the solution increases after the amendment is added, weakening the thickness of the soil particle binding water diffusion layer and improving the soil performance. With the growth and development of the strength of mica schist improved soil, a series of chemical reactions occur among several solidification components of the new type of improver. Various chemical reactions interact, correlate, and promote each other. The main reaction equations are as follows:

Al_2_O_3_ + Ca(OH)_2_ + H_2_O → xCaO·yAl_2_O_3_·zH_2_O (1)

SiO_2_ + Ca(OH)_2_ + H_2_O → xCaO·ySiO_2_·zH_2_O (2)

Al_2_O_3_ + Ca(OH)_2_ + SO_4_^2−^ + H_2_O → xCaO·yAl_2_O_3_·zCaSO_4_·mH_2_O (3)

Al_2_O_3_ + SiO_2_ + Ca(OH)_2_ + H_2_O → xCaO·yAl_2_O_3_·zSiO_2_·mH_2_O (4)

Cement contains C_2_S (dicalcium silicate) and C_3_S (tricalcium silicate) which generate a large amount of Ca(OH)_2_ when they undergo hydration reactions [34], providing the alkaline environment required for related chemical reactions. The surface of soil particles usually adsorbs a large number of low-valence cations such as Na^+^, K^+^, and H^+^. These cations can conduct ion exchange interactions with high-valence cations such as Ca^2+^ in the solution [35], reduce the double electric layer structure of particles, reduce the electrokinetic potential, release the bound water, reduce the thickness of the bound water film on the particle surface, enhance the agglomeration, accelerate the aggregation of soil particles, and cause loose soil particles to evolve into a granular structure, thus improving the integrity of the soil mass. Fly ash and slag contain a large amount of active SiO_2_ and active Al_2_O_3_ [36]. Under the activation of the alkali environment, volcanic ash reaction takes place to generate amorphous gel such as C-S-H, C-A-H, and C-A-S-H [37], enhancing the cementation performance between particle interfaces. The addition of desulfurized gypsum promotes the growth of expansive crystals of ettringite [38]. The soil system becomes denser due to the expansion of fibrous crystals. As the solidification reaction of the improved material in the improved soil system proceeds, the water content in the solidified soil continues to decrease. The incomplete chemical composition further refines the soil pore structure and plays a role in filling with microaggregates. The early strength of solidified soil is mainly provided by the hydration reaction of cement components, and the later strength is provided by the pozzolanic reaction of solid waste components. The pozzolanic reaction rate of solid waste is slow, which explains the test results that the unconfined compressive strength of solidified soil develops rapidly in the early stage and slowly in the later stage [39]. Due to the decrease in water content in the soil, the chemical reaction rate that promotes soil hardening gradually slows down and tends to flatten out with the increase in improved soil solidification time. This also indicates that the mechanical strength of solidified soil samples increases rapidly in the early stage, while the later stage increases slowly.

## 5. Conclusions

This article uses cement–fly ash–slag–desulfurization gypsum to develop a new type of fly ash solidification agent, CFSD, to cure mica schist weathered soil. Through mechanical tests, microscopic tests, and microscopic mechanism models, the solidification mechanism of the CFSD solidification agent for mica schist weathered soil is revealed. The main research contents and conclusions of this paper are as follows:

(1) The mechanical test reveals that the CFSD curing agent can effectively improve the mechanical properties of mica schist weathered soil, and the failure strength of the solidified soil specimen can reach 1.362 MPa after 7 days of curing with the addition of 4%. With the increase in the amount of CFSD curing agent and the curing age, the uniaxial stress–strain curve steepens, and the brittleness of the solidified soil shows an increasing trend. The failure strain and failure deviator stress of solidified soil show a positive growth trend with the increase in dosage, and the curve develops from the strong softening type to the weak softening type with the increase in the initial effective confining pressure.

(2) The microquantitative analysis of the cured soil based on SEM images combined with Image J shows that, with an increase in curing agent dosage and curing age, the microstructure of the cured soil is improved, the percentage of large pore size intervals decreases from 56.46% to 27.92%, the porosity decreases from 12.51% to 4.6%, and the shape of the pores evolves gradually from sub-circular macropores to bar-shaped micropores.

(3) The synergistic effect between the chemical components of the curing agent is the main source of strength of CFSD-cured soil. The curing agent produces a volcanic ash reaction in an alkaline environment; the generation of swelling hydrate AFt as a “skeleton” effectively fills the pore space and supports the neighboring soil particles; the C-A-H, C-S-H gel, and the soil particles bond to the soil particles, which together constitute the gel–crystal network structure; the pore structure and skeleton are strengthened, and the macromechanical behavior is manifested as the mica schist soil curing soil specimens with strengthened mechanical properties.

## Figures and Tables

**Table 1:** Basic physical properties of mica schist strongly weathered soil.

Liquid Limit (%)	Plastic Limit (%)	Plasticity Index (%)	Maximum Dry Density (g/cm^3^)	Optimum Eater Content (%)	Free Swelling Rate (%)
37	21	16	1.97	10	21

**Table 2:** Main chemical components of curing agent (%).

Chemical Composition	SiO_2_	Al_2_O_3_	CaO	MgO	Fe_2_O_3_	SO_3_
Cement	20.53	6.05	61.22	3.52	3.07	2.11
Fly ash	62.25	21.67	2.05	2.17	6.21	0.85
Slag	28.49	18.03	32.54	9.49	0.33	2.49
Desulfurization gypsum	30.22	16.02	41.66	8.22	0.43	1.45

**Table 3:** Test data.

Test Piece	Strain/%	Stress/MPa
4%-7 d	2.814	1.362
8%-7 d	1.909	2.433
13%-7 d	1.389	2.997
4%-28 d	2.393	2.768
8%-28 d	1.452	3.709
13%-28 d	1.107	4.805
4%-90 d	2.241	3.11
8%-90 d	1.406	4.563
13%-90 d	0.938	5.936

**Table 4:** Test data.

Test Piece	Strain/%	Stress/kPa
4%-50 kPa	3.28	210.29
4%-100 kPa	4.04	295.25
4%-200 kPa	5.25	465.27
4%-300 kPa	6.26	635.28
8%-50 kPa	3.69	278.35
8%-100 kPa	4.82	376.76
8%-200 kPa	6.25	573.83
8%-300 kPa	8.63	770.5
13%-50 kPa	4.04	304.38
13%-100 kPa	5.52	405.54
13%-200 kPa	7.36	607.63
13%-300 kPa	9.71	809.83

## Data Availability

Not applicable.
